# Inactivation of Anandamide Signaling: A Continuing Debate

**DOI:** 10.3390/ph3113355

**Published:** 2010-10-28

**Authors:** Hesham Khairy, Wael E. Houssen

**Affiliations:** School of Medical Sciences, Institute of Medical Sciences, University of Aberdeen, Aberdeen AB25 2ZD, Scotland, UK; E-Mail: khairyhesham@yahoo.com (H.K.)

**Keywords:** endocannabinoid inactivation, anandamide, ethanolamine

## Abstract

Since the first endocannabinoid anandamide was identified in 1992, extensive research has been conducted to characterize the elements of the tightly controlled endocannabinoid signaling system. While it was established that the activity of endocannabinoids are terminated by a two-step process that includes cellular uptake and degradation, there is still a continuing debate about the mechanistic role of these processes in inactivating anandamide signals.

## 1. Introduction

The medical use of *Cannabis sativa* L. is as ancient as the two thousand years old Chinese treatise that described the use of the female plant as an anesthetic in surgery by using its resin mixed with wine [1]. The term “cannabinoids” covers a wide range of compounds derived from the Cannabis plant. The first attempt to identify a cannabinoid was reported by Wood *et al*., who isolated cannabinol (CBN) from a red oily extract of *Cannabis* [2], while 60 years later, the structures of the cannabidiol (CBD) and the psychoactive compound (−)-trans-∆^9^-tetrahydrocannabinol (∆^9^-THC) were reported by Mechoulam’s laboratory [3]. To date, there are over 65 cannabinoids with various medical applications including treatment of multiple sclerosis and obesity, pain relief and cancer chemotherapy [4,5,6,7]. 

**Figure 1:**
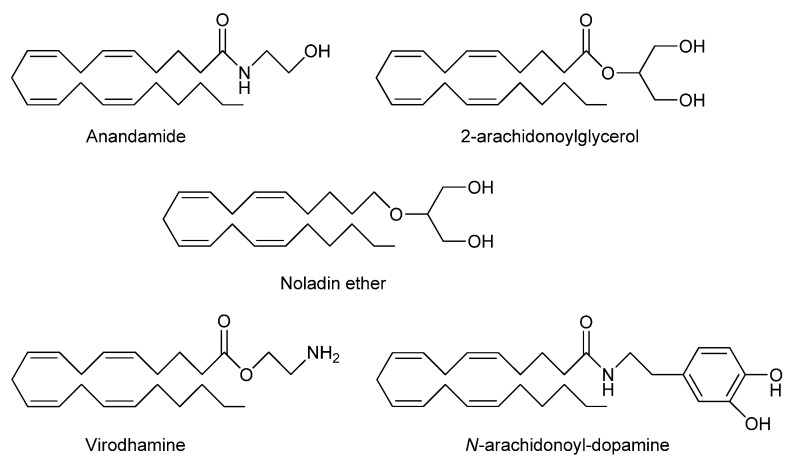
Chemical structures of proposed endocannabinoids.

The identification of cannabinoid CB1 [8,9] and CB2 receptors [10] was the trigger for rapid identification of their natural endogenous ligands known as endocannabinoids. These are lipid inter-cellular signaling molecules derived from arachidonic acid conjugated with glycerol or ethanolamine and include anandamide, also known as *N*-arachidonylethanolamine (anandamide, AEA) [11] and 2-arachidonoylglycerol (2-AG) [12]. More recently-discovered endogenous cannabinoids include noladin ether (2-arachidonoylglycerolether) [13], *N*-arachidonoyl-dopamine (NADA) [14] and virodhamine [15] (Figure 1) although AEA and 2-AG are the only detectable endocannabinoids so far in human plasma using LCMS analysis [16].

The present review gives a general view of the endocannabinoid system and focuses on the inactivation of anandamide signaling processes in the light of extensive research that has been aimed at addressing many of the questions regarding the anandamide reuptake and degradation processes as effective mechanisms for terminating endocannabinoid actions.

## 2. Endocannabinoid System

The endocannabinoid system is a lipid signaling system which has important regulatory functions throughout the body in all vertebrates. It consists of endocannabinoid ligands along with their receptors, signaling pathways and cellular machinery for their biosynthesis [17], uptake [18] and degradation [19]. The present review will focus on AEA as its pharmacological activities and metabolism has been more extensively investigated compared with other recently discovered and emerging endocannabinoids.

Furthermore, three fatty acid-derived compounds were found to function in concert with endocannabinoids despite having no affinity for cannabinoid receptors and, thus, known as endocannabinoid congeners. These compounds are: (**A**) *N*-palmitoylethanolamine (PEA), a well known anti-inflammatory compound which was found in lipid extracts of various natural products. It was shown to enhance the endocannabinoid actions on cannabinoid receptors by interfering with their inactivation [20]; (**B**) oleamide, a primary fatty acid amide that is accumulated in the cerebrospinal fluid (CSF) of sleep–deprived cats, was found to be degraded by fatty acid amide hydrolase (FAAH). Its systemic administration produced cannabimimetic effects [21]; (**C**) *N*-arachidonoyl amino acids, formed by conjugation of arachidonic acid with various amino acids including, glycine, alanine, valine, cysteine and serine [22]. These compounds act as endogenous modifiers of endocannabinoid activities through indirect entourage effects on endocannabinoid degradation and/or reuptake [23]. However, the effects of these compounds *in vivo* remain to be determined. 

**Figure 2:**
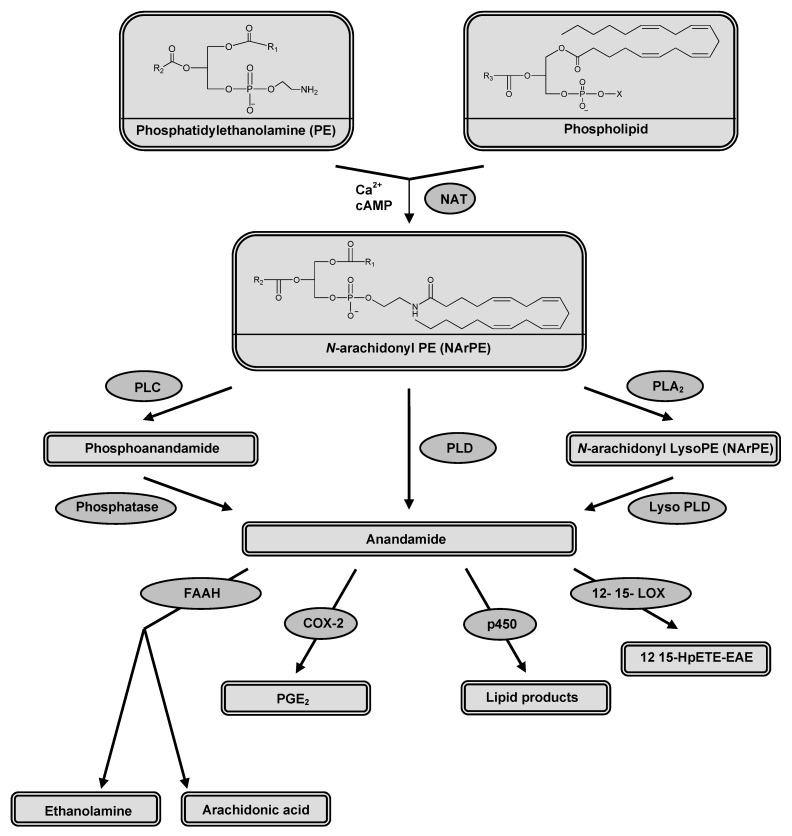
Enzymes involved in AEA synthesis; *N*-acyltransferase (NAT), protein tyrosine phosphatase (PLP) and phospholipase D (PLD) and in AEA degradation; fatty acid amide hydrolase (FAAH), cyclooxygenase-2 (COX-2), cytochrome P450, and 12-, 15-lipo-oxygenase (LOX).

### 2.1. Synthesis and release

Anandamide is synthesized (Figure 2) by stimulus-dependent cleavage of its precursor, *N*-arachidonoyl-phosphatidylethanolamine (NArPE). NArPE is formed by the enzyme *N*-acyltransferase (NAT) in the presence of calcium. Cyclic AMP was found to enhance the activity of NAT through activating protein kinase A [24]. AEA is released from NArPE by cleavage of phosphodiester bond catalyzed by a phospholipase D (PLD), a member of metallo-β-lactamase family [25]. Other alternative pathways for AEA synthesis have been also reported [26,27,28].

Many factors contribute to anandamide release from the plasma membrane. Neuronal activity (*i.e.,* depolarization) produces Ca^2+^-dependent anandamide release. The anandamide then diffuses laterally in the cell membrane to exert its effects on targeted receptors and/or ion channels [29]. Alternatively, anandamide could be released into the extracellular fluid and subsequently bind to protein carrier as albumin to reach a distant targets at presynaptic terminals [30].

### 2.2. Functions and actions

In order to characterize the exact meaning of the term “inactivation”, we should outline briefly the functions and actions of endocannabinoid system. The tissue distribution of different types of cannabinoid receptor accounts for the diversity of endocannabinoid effects either centrally or peripherally. Extensive studies had been conducted to address the possible physiological functions of the endocannabinoid system. Generally speaking, endocannabinoids are produced on demand and act as local mediators that control the function of the secretory cells *i.e.,* autocrine, or the adjacent cells *i.e.,* paracrine [31]. These actions vary according to the targeted receptor. Thus, CB1 receptors mediate the neuromodulatory action regulating pain perception and food intake [5,32], as well as, cardiovascular, respiratory and gastrointestinal effects [33,34,35], while CB2 receptors mediate the humoral immune modulation [36].

Cannabinoid receptor signaling is mainly coupled to G_i/o_ proteins with consequent inhibition of adenylyl cyclase and PKA along with stimulation of mitogen-activated protein kinase (MAPK) that regulates nuclear transcription factors [37,38]. These effects are probably mediated by the free β/γ G-protein subunit dimers although there remains some uncertainty about the subtype of G_i/o_ proteins that might be associated with the responses [39]. It is thought that the same mechanism is responsible for activation of phosphoinositide-3-kinase (PI-3-K). Active PI-3-K phosphorylate the inositol lipids and with subsequent activation of Raf-1 protein kinase [40]. Activation of CB2 receptors modulates the cytokine production and the migration of the immune cells thus influencing the immune response [41].

Anandamide has been found to inhibit high voltage-activated calcium currents but at least part of this effect is insensitive to CB1 receptor inhibition suggesting multiple mechanisms of high voltage-activated calcium currents (VACC) modulation [42,43]. Apart from activation of cannabinoid receptors, anandamide was found to produce direct inhibition of Shaker-related Kv1.2 channels, an action that was insensitive to either pertussis toxin or CB1 cannabinoid receptor antagonist, SR141716 [44]. Additionally, anandamide was reported to inhibit the delayed rectifier component of the potassium current in smooth muscle preparation from rat aorta in a cannabinoid receptor-independent way [45]. 

## 3. Reuptake

Anandamide movement across the cell membrane is still a matter of considerable debate being either mediated through the concentration gradient (*i.e.,* simple diffusion) or through specific transporter machinery (*i.e.,* facilitated diffusion). Being lipophilic in nature, anandamide rapidly accumulates inside the cell through a temperature-sensitive, ATP-independent, saturable mechanism that could be inhibited by several analogues [46,47,48,49]. There are also pieces of evidence that suggest the presence of a carrier protein, anandamide membrane transporter (AMT) that bind and translocates anandamide in both directions across the cell membrane. These findings were confirmed by the systemic administration of LY2318912, a competitive inhibitor of anandamide uptake, which elevates anandamide level 5-fold in the brain [50]. On the other hand, there is also much evidence to suggest that anandamide movement across the cell occurs by simple diffusion along the concentration gradient enhanced by rapid intracellular hydrolysis of anandamide catalyzed by (FAAH). This hydrolysis mechanism could be responsible for the apparent saturability of anandamide transport [51,52,53]. These suggestions were enforced by finding that the inhibiting effects of LY2318912 on AEA reuptake were mediated through FAAH inhibition that in turn reduces the concentration gradients necessary for AEA movement across the cell membrane [54,55]. Moreover, temperature was found to affect to a greater extent the concentration of free anandamide rather than affecting the reuptake process itself [56]. It was also found that the intracellular concentration of anandamide is 1,000-fold its concentration in the extracellular compartment at equilibrium in the cerebellar granule neurons [57]. This could be explained by the intracellular reversible sequestration of anandamide via its association with either membranous compartments (e.g. endoplasmic reticulum) [58] or an intracellular protein [59] leaving only a small fraction of free anandamide. This sequestration could explain apparent saturation of putative transport processes and selective inhibition of anandamide accumulation by related lipophilic molecules [60]. Additionally, the AEA uptake inhibitor, AM404, was thought to provide evidence for the existence of AMT [18]. Later on, it was demonstrated that AM404 is not selective for uptake inhibition but interferes with anandamide hydrolysis by acting as a substrate for FAAH [61]. 

Parallel to the debate about the existence of AMT, there is another debate about the role of AEA uptake in terminating AEA activity. The endocannabinoid anandamide was found to activate transient receptor potential vanilloide receptor type 1 (TRPV1) on the perivascular sensory nerves an activity that subsequently produced vasodilatation of associated vascular smooth muscle [62]. Furthermore, it was demonstrated that AEA acts as a full agonist for TRPV1 receptors in recombinant expression systems. Anandamide also shows low affinity for TRPV1 receptors but this could be enhanced by palmitoylethanolamide [63]. In cultured DRG neurones, the AEA effects on TRPV1 were found to inhibit VACC and enhance Ca^2+^ influx. These effects were best achieved via intracellular delivery of AEA rather than its extracellular application [42]. Anandamide-induced intracellular activation of TRPV1 receptor leads to increased calcium influx through TRPV1 channels in transfected cells and DRG neurones. This might occur prior to activation of cannabinoid receptors and could be related to anandamide control over transmitter release [64]. Therefore, influencing the activity of the putative AMT might subsequently affect the TRPV1-mediated response of anandamide [65].

In rat DRG neurones, TRPV1 channels were activated and/or sensitized by anandamide through alteration of protein kinase C (PKC) and protein kinase A (PKA) activities [66,67,68]. PKC directly phosphorylated the amino acid residues in TRPV1 channels resulting in their sensitization and channel opening [69]. Along with PKC stimulation, the relief of tonic inhibition of phosphatidyl-inositol-bisphosphate (PIP_2_) sensitizes TRPV1 channels. Much evidence suggests roles for inflammatory mediators such as nerve growth factor (NGF) and bradykinin in regulating both PKC and PIP2 signaling [70]. It was found that NGF levels in the medium influence TRPV1 receptor function in cultured DRG neurons. Specifically, high levels of NGF (200 nM compared with 20 nM) increased the population of neurones in which AEA activates a current carried through TRPV1 receptors. This appears to be specific for this type of signaling since AEA-mediated inhibition of voltage-activated Ca^2+^ channels in DRG neurones was not enhanced by high NGF in the culture medium [71].

Natural co-expression of CB1 receptors and TRPV1 receptors was demonstrated in DRG neurones, spinal cord, myenteric neurones and rat brain [72,73,74] with suspected functional cross-talk especially for AEA and NADA, the endocannabinoids that activate both receptor types. If these compounds originated from outside the cell, they would be predicted to activate CB1 receptors first then TRPV1 activation occurs after cellular uptake. On the other hand, if these compounds were synthesized inside the cell, they would activate TRPV1 channels prior to CB1 receptor activation. In either condition, switching between cannabinoid and vanilloid receptor activation occurs upon transport of these compounds across the cell membrane. For more details about CB1 and TRPV1 cross talk see [71,75,76].

## 4. Hydrolysis

In 1993, it was first reported that anandamide hydrolysis into free arachidonic acid and ethanolamine is catalyzed by FAAH [77]. The brain anandamide levels were found to be 15-fold higher due to low anandamide hydrolytic activity in FAAH-knockout mice when compared to the wild type animals [78]. FAAH is a membrane-bound protein that is widely distributed in the liver, brain, small intestine and many other organs [19,79,80]. Using immunohistochemical localization, it was illustrated that FAAH distribution in the brain was heterogeneous, mostly complementary with CB1 cannabinoid receptor [81] and preferentially located in large neurones such as pyramidal cells in the cerebral cortex and hippocampus [82]. The presence of FAAH on the endoplasmic reticulum, away from cannabinoid receptors located on the cell membrane, requires the presence of an intracellular carrier protein. This carrier overcomes the low solubility of AEA in the hydrophilic cytosol and facilitates trafficking from the site of AEA signaling to its primary site of catabolism. Recent studies have revealed the contribution of fatty acid binding proteins (FABPs) as intracellular AEA trafficking proteins [83]. FAAH could be inhibited either reversibly by trifluoromethyl ketonase (TFMK) [84] or irreversibly by phenylmethylsulfonylflouride (PMSF) [85] and URB597 [86] with the resulting elevation of the endocannabinoid anandamide. FAAH inhibition, thus, provides a therapeutic alternative to direct cannabinoid receptor agonists with added specificity because of the “on demand” biosynthesis of anandamide [87]. Novel FAAH inhibitors, PF-750 and PF-622, showed more potent and selective inhibition of FAAH in a time-dependent manner [88]. In preclinical studies, URB597 was found to produce analgesic, anxiolytic-like and antidepressant-like effects in rodents, which are not accompanied by overt signs of abuse liability. There is evidence that the drug offers a possible therapeutic avenue for the treatment of cannabis withdrawal [89]. Moreover, the effect of dual blockage of FAAH and monoacylglycerol lipase (MAGL; the enzyme that hydrolyze 2-AG), by using JZL195 was found to mimic the effect of direct CB1 agonists. This contrasts to the outcome of selectively blocking either enzyme alone [90]. Recently, FAAH-2 was identified as a second FAAH enzyme that hydrolyzes AEA with activity ~30% of that FAAH in intact cells. In contrast to FAAH, FAAH-2 was localized on lipid droplets that represent novel sites for AEA and *N*-aclyethanolamine (NAE) inactivation [91]. It should be noted that FAAH-2 has a limited species distribution and so plays no role in investigations of endocannabinoid turnover in mice and rats. Recently, a new isozyme, *N*-acylethanolamine-hydrolyzing acid amidase (NAAA), was detected in several macrophage-like cells and showed AEA-degradation activity in acidic pH [92,93]. 

**Figure 3:**
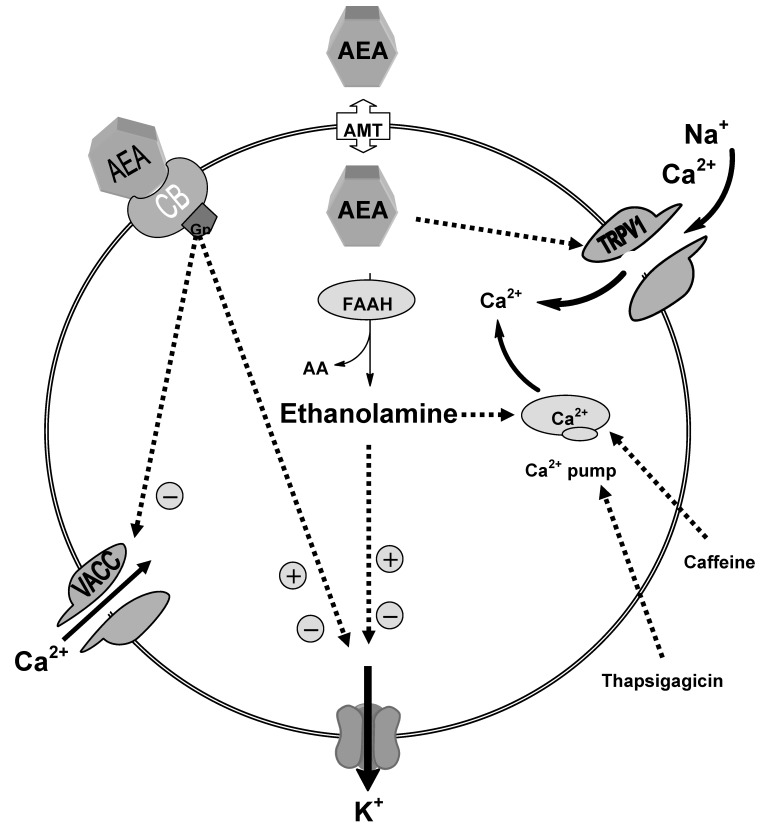
Schematic diagram showing the possible mechanisms of ethanolamine actions; our data suggested that enhancement of the intracellular Ca^2+^ level produced by ethanolamine is mediated through intracellular signalling mechanism influencing the thapsigargicin and caffeine-sensitive stores. Ethanolamine modulation of the potassium conductance is mediated independently from increased intracellular calcium.

Several studies had revealed the activity of the downstream metabolites of AEA. Our studies indicated that ethanolamine is active but is modulated by distinct mechanisms, intracellular signaling via thapsigargicin and caffeine-sensitive stores and voltage-activated K^+^ currents in DRG neurons from neonatal rats (Figure 3) [94]. As with the actions of AEA on DRG neurons, it is difficult to predict the overall action of ethanolamine on neuronal excitability and synaptic transmission. A combination of K^+^ current inhibition and slowed action potential repolarization with enhanced intracellular Ca^2+^ signaling would be predicted to at least initially to enhance neurotransmission. In contrast, sustained large increases in voltage-activated K^+^ currents might be expected to shorten action potentials and reduce neuronal excitability. Both of these possible scenarios are consistent with the actions of AEA [43] and provide what has previously been described as a capacity for contradictory or reinforcing pathways [95].

Our data also revealed that ethanolamine enhancement of the intracellular Ca^2+^ flux was also found to be unaffected either in Pertussis toxin (PTX)-treated neurons or with the continual application of CB1 antagonist, SR141716 which adds weight to the hypothesis that these effects are not mediated through the CB1 receptor signaling pathway involving the inhibitory G-proteins. Moreover, ethanolamine modulation of voltage-activated K^+^ currents was found to occur independently from enhancing intracellular Ca^2+^ levels. The evidence for this was the persistence of modulations of voltage-activated K^+^ currents, firstly, in Ca^2+^ free media and secondly, in the continual presence of thapsigargicin. In line with our findings, it was previously found that AEA-inhibitory actions on voltage-activated K^+^ current occurred independently from the inhibition of VACC [42,43]. Similarly, we found that ethanolamine had no effect on the voltage-activated calcium current but modulated the voltage-activated K^+^ current. These findings enforce the possibility that while modulating effects of ethanolamine on voltage-activated K^+^ current could be mediated through the same anandamide signaling pathway, the increased intracellular Ca^2+^ effects are mediated via intracellular signaling mechanism modulating the calcium store. These findings are in agreement with the previous study [43] that pointed out the potential role of active, downstream metabolites of anandamide. It was found that irreversible blocking of FAAH by phenylmethanesulfonyl fluoride (PMSF), thus preventing the hydrolysis of AEA to its downstream metabolites, attenuated the actions of AEA on potassium conductance. This finding was supported by the inactivity of hydrolysis-resistant methanandamide in modulating potassium current. 

With regard to the other downstream metabolites of AEA, arachidonic acid (AA) is found primarily in an esterified form with the membrane phospholipids and is released by the actions of phospholipases. Arachidonic acid can be metabolized by three enzymatic pathways: the lipoxygenase pathway forming leukotrienes and lipoxins, the cyclooxygenase pathway (COX) producing prostaglandins and thromboxanes, and the cytochrome P450 (cP450) pathway generating epoxygenated products [96]. Aspirin and other non-steroidal anti-inflammatory drugs (NSAIDs) act through inhibiting AA oxidation by COX enzymes thus inhibiting he formation of downstream metabolites of AA. Prostaglandin E_2_ has been shown to increase the sensitivity to noxious stimuli via increasing the excitability of sensory neurones [97]. Additionally, it was found that some endogenous cytochrome P450 arachidonic acid metabolites activate cannabinoid receptors CB1 and CB2 in brain, kidney and spleen with high affinity and elicited biological response *in vivo* and *in vitro* in cultured cells expressing CB receptors although AA by itself failed to activate either CB1 or CB2 receptors [98].

## 5. Oxidation

In 1997 the oxidative pathway of anandamide mediated via cyclooxygenase-2 (COX-2) was identified [99]. This pathway generated prostaglandin E_2_-ethanolamide (PGE_2_-EA) which is resistant to hydrolysis and does not appear to activate prostanoid receptors, or interact with FAAH or activate TRPV1 receptors [100]. These findings made the COX-2 metabolism of AEA unclear in terms of biological significance. To date, AEA oxidation metabolites have not been detected in native tissue, yet growing evidence has referred to the enhanced role of the oxidative metabolic pathways when the endocannabinoid system is activated while FAAH activity is suppressed [101]. Oxidation of AEA can be also mediated via lipooxygenase (LOX) enzyme producing 12- and 15-HpETE-EA both *in vitro* and *in vivo* [102,103]. These metabolites are still active at the cannabinoid receptors, although less effective than the parent compound (AEA), adding another question about the biological relevance of such reactions. In FAAH inactivated animals an alternative pathway of AEA catabolism was identified through detecting the presence of *O*-phosphorylcholine-NAE in the CNS tissue. The *O*-phosphorlycholine-NAE could be hydrolysed back to its parent compound AEA allowing a biochemical route for storing and release of AEA and its congeners rather than being a secondary pathway for AEA inactivation [104,105].

## 6. Concluding Remarks

While it is well established that the anandamide signaling is tightly controlled by the inactivation processes including re-uptake and degradation via either hydrolysis or oxidation, the efficacy of such processes in terminating AEA actions is still a matter of debate. Anandamide re-uptake could be viewed as a switching mechanism from one receptor, CB1, to another, TRPV1. While the metabolites of anandamide degradation showed various intracellular activities which could either potentiate or counteract anandamide actions. Thus, it is important to characterize the predominant factor that could be varied from one cell to another according to the distribution of endocannabinoid-targeted receptors and degradation enzymes. Proper characterization of tissue-specific inactivation could direct endocannabinoid-therapeutic strategies to more specific actions and minimize the side effects. Moreover, future studies may consider other ways of terminating AEA actions such as down regulation and/or desensitization of the endocannabinoid-targeted receptors.
